# Crystal structure of 1-{2-[(2-meth­oxy­phen­yl)selan­yl]phen­yl}-4-phenyl-1*H*-1,2,3-triazole

**DOI:** 10.1107/S2056989015003230

**Published:** 2015-02-25

**Authors:** Leandro R. S. Camargo, Julio Zukerman-Schpector, Anna M. Deobald, Antonio L. Braga, Edward R. T. Tiekink

**Affiliations:** a Departmento de Química, Universidade Federal de São Carlos, 13565-905 São Carlos, SP, Brazil; b Departmento de Química, Universidade Federal de Santa Maria, 97105-900 Santa Maria, RS, Brazil; c Departmento de Química, Universidade Federal de Santa Catarina, 88040-900 Florianópolis, SC, Brazil; d Department of Chemistry, University of Malaya, 50603 Kuala Lumpur, Malaysia

**Keywords:** crystal structure, organoselenium, Se⋯O halogen bonding, hydrogen bonding, C—H⋯π inter­actions

## Abstract

In the title compound, C_21_H_17_N_3_OSe, the dihedral angles between the central five-membered ring and the C- and N-bound rings are 17.89 (10) and 42.35 (10)°, respectively, indicating the mol­ecule is twisted. The dihedral angle between the Se-bound rings is 85.36 (10)°. A close intra­molecular Se⋯O contact of 2.8507 (13) Å is noted. In the crystal, C—H⋯O, C—H⋯N and C—H⋯π inter­actions lead to the formation of supra­molecular layers parallel to (011); these stack with no specific inter­molecular inter­actions between them.

## Related literature   

For background to aryl­seleno-1,2,3-triazoles and to the synthesis of the title compound, see: Deobald *et al.* (2011 ▸). For an analysis of intra- and inter­molecular Se⋯O inter­actions, see: Linden *et al.* (2014 ▸). For a related organoselenium compound with a 1,2,3-triazole residue, see: Camargo *et al.* (2015 ▸).
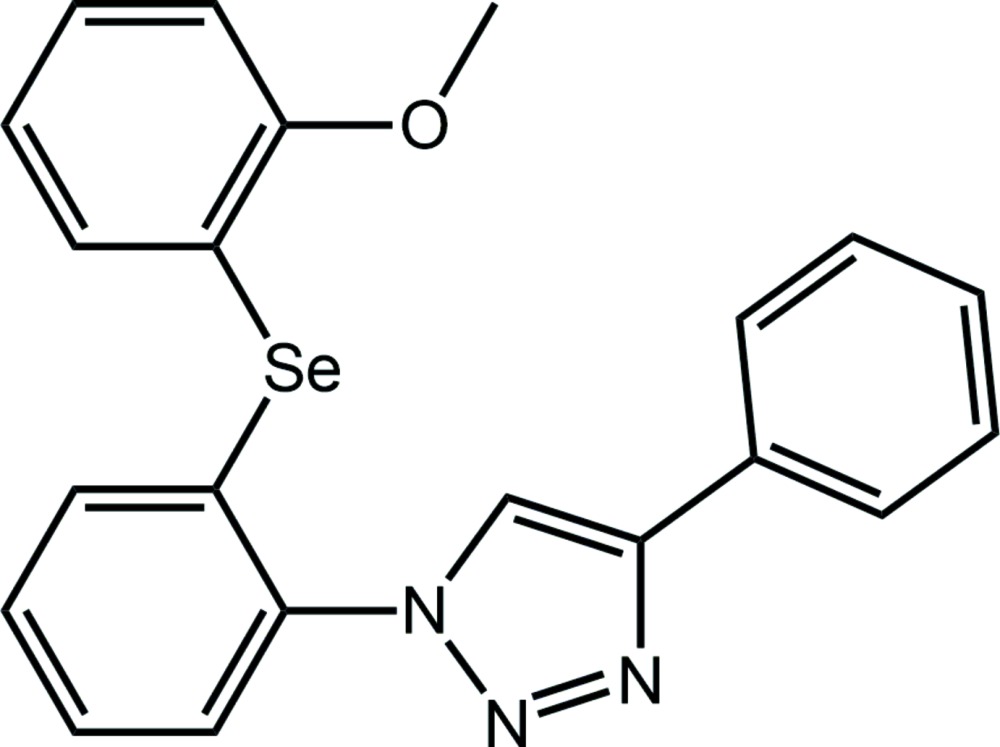


## Experimental   

### Crystal data  

C_21_H_17_N_3_OSe
*M* *_r_* = 406.33
Triclinic, 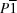

*a* = 5.6565 (3) Å
*b* = 10.3682 (5) Å
*c* = 15.3358 (7) Å
α = 81.604 (4)°
β = 80.006 (4)°
γ = 85.340 (4)°
*V* = 874.83 (8) Å^3^
*Z* = 2
Mo *K*α radiation
μ = 2.16 mm^−1^
*T* = 100 K
0.30 × 0.20 × 0.10 mm

### Data collection  

Agilent SuperNova CCD diffractometer
Absorption correction: multi-scan (*CrysAlis PRO*; Agilent, 2011 ▸) *T*_min_ = 0.759, *T*_max_ = 1.000
6845 measured reflections
3869 independent reflections
3548 reflections with *I* > 2σ(*I*)
*R*_int_ = 0.040

### Refinement  

*R*[*F* ^2^ > 2σ(*F* ^2^)] = 0.028
*wR*(*F* ^2^) = 0.064
*S* = 1.01
3869 reflections
236 parameters
H-atom parameters constrained
Δρ_max_ = 0.41 e Å^−3^
Δρ_min_ = −0.52 e Å^−3^

### 

Data collection: *CrysAlis PRO* (Agilent, 2011 ▸); cell refinement: *CrysAlis PRO*; data reduction: *CrysAlis PRO*; program(s) used to solve structure: *SIR2014* (Burla *et al.*, 2015 ▸); program(s) used to refine structure: *SHELXL2014* (Sheldrick, 2015 ▸); molecular graphics: *ORTEP-3 for Windows* (Farrugia, 2012 ▸) and *DIAMOND* (Brandenburg, 2006 ▸); software used to prepare material for publication: *MarvinSketch* (ChemAxon, 2010 ▸) and *publCIF* (Westrip, 2010 ▸).

## Supplementary Material

Crystal structure: contains datablock(s) I, New_Global_Publ_Block. DOI: 10.1107/S2056989015003230/hg5432sup1.cif

Structure factors: contains datablock(s) I. DOI: 10.1107/S2056989015003230/hg5432Isup2.hkl

Supporting information file. DOI: 10.1107/S2056989015003230/hg5432Isup3.cml

**Fig. 1.**
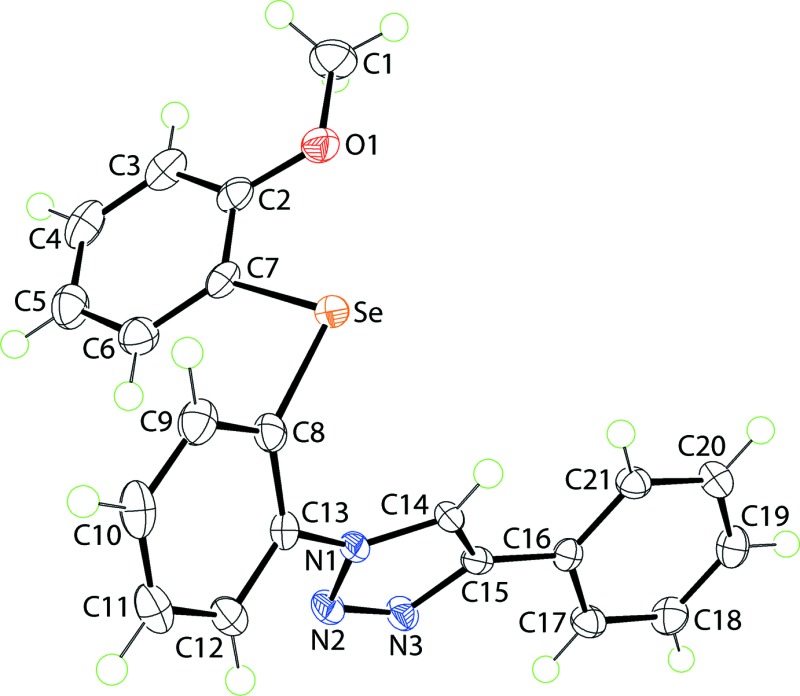
The molecular structure of the title compound showing the atom-labelling scheme and displacement ellipsoids at the 70% probability level.

**Fig. 2.**
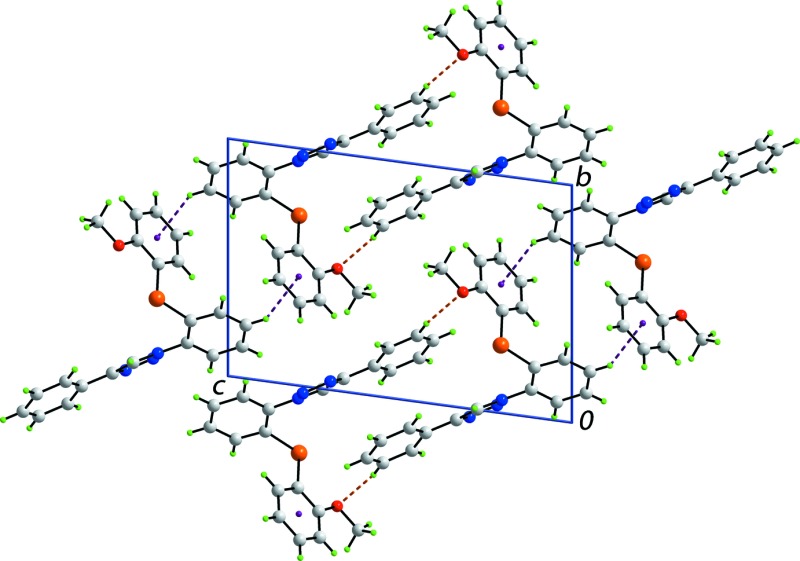
A view in projection down the a axis of the unit-cell contents. The C—H···O, C—H···N and C—H···π interactions are shown as orange, blue and purple dashed lines, respectively.

. DOI: 10.1107/S2056989015003230/hg5432fig1.tif
The mol­ecular structure of the title compound showing the atom-labelling scheme and displacement ellipsoids at the 70% probability level.

a . DOI: 10.1107/S2056989015003230/hg5432fig2.tif
A view in projection down the *a* axis of the unit-cell contents. The C—H⋯O, C—H⋯N and C—H⋯π inter­actions are shown as orange, blue and purple dashed lines, respectively.

CCDC reference: 1049507

Additional supporting information:  crystallographic information; 3D view; checkCIF report

## Figures and Tables

**Table 1:** Hydrogen-bond geometry (, ) *Cg*1 is the centroid of the C1C6 ring.

*D*H*A*	*D*H	H*A*	*D* *A*	*D*H*A*
C18H18O1^i^	0.95	2.54	3.472(2)	165
C14H14N3^ii^	0.95	2.58	3.520(2)	170
C10H10*Cg*1^iii^	0.95	2.82	3.630(2)	144

Symmetry codes: (i) 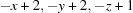; (ii) 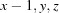; (iii) 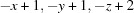.

## supplementary crystallographic information

### S1. Experimental

The compound was prepared in accord with the literature (Deobald *et al.*, 2011). Crystals were obtained by taking 200 mg of sample into a sample vial containing methanol (10 ml) and letting it stand at room temperature.

### S2. Refinement

Carbon-bound H-atoms were placed in calculated positions (C—H = 0.95 to 0.98 Å) and were included in the refinement in the riding model approximation, with *U_iso_*(H) = 1.2–1.5*U_eq_*(C).

### Crystal data

**Table:**

C_21_H_17_N_3_OSe	*Z* = 2
*M**_r_* = 406.33	*F*(000) = 412
Triclinic, *P*1	*D*_x_ = 1.543 Mg m^−^^3^
*a* = 5.6565 (3) Å	Mo *K*α radiation, λ = 0.71073 Å
*b* = 10.3682 (5) Å	Cell parameters from 4524 reflections
*c* = 15.3358 (7) Å	θ = 2.6–29.2°
α = 81.604 (4)°	µ = 2.16 mm^−^^1^
β = 80.006 (4)°	*T* = 100 K
γ = 85.340 (4)°	Prism, colourless
*V* = 874.83 (8) Å^3^	0.30 × 0.20 × 0.10 mm

### Data collection

**Table:**

Agilent SuperNova CCD diffractometer	3548 reflections with *I* > 2σ(*I*)
Radiation source: SuperNova (Cu) X-ray Source	*R*_int_ = 0.040
ω scans	θ_max_ = 27.5°, θ_min_ = 2.6°
Absorption correction: multi-scan (*CrysAlis PRO*; Agilent, 2011)	*h* = −6→7
*T*_min_ = 0.759, *T*_max_ = 1.000	*k* = −13→12
6845 measured reflections	*l* = −19→19
3869 independent reflections	

### Refinement

**Table:**

Refinement on *F*^2^	0 restraints
Least-squares matrix: full	Hydrogen site location: inferred from neighbouring sites
*R*[*F*^2^ > 2σ(*F*^2^)] = 0.028	H-atom parameters constrained
*wR*(*F*^2^) = 0.064	*w* = 1/[σ^2^(*F*_o_^2^) + (0.026*P*)^2^ + 0.4456*P*] where *P* = (*F*_o_^2^ + 2*F*_c_^2^)/3
*S* = 1.01	(Δ/σ)_max_ = 0.002
3869 reflections	Δρ_max_ = 0.41 e Å^−^^3^
236 parameters	Δρ_min_ = −0.52 e Å^−^^3^

### Special details

**Table:**

Geometry. All e.s.d.'s (except the e.s.d. in the dihedral angle between two l.s. planes) are estimated using the full covariance matrix. The cell e.s.d.'s are taken into account individually in the estimation of e.s.d.'s in distances, angles and torsion angles; correlations between e.s.d.'s in cell parameters are only used when they are defined by crystal symmetry. An approximate (isotropic) treatment of cell e.s.d.'s is used for estimating e.s.d.'s involving l.s. planes.

### Fractional atomic coordinates and isotropic or equivalent isotropic displacement parameters (Å^2^)

**Table:**

	*x*	*y*	*z*	*U*_iso_*/*U*_eq_	
Se	0.46182 (3)	0.71551 (2)	0.79326 (2)	0.01503 (7)	
O1	0.5130 (2)	0.51326 (12)	0.68268 (9)	0.0185 (3)	
N1	0.8216 (3)	0.94528 (14)	0.79605 (10)	0.0128 (3)	
N2	1.0599 (3)	0.96928 (15)	0.78243 (11)	0.0154 (3)	
N3	1.1085 (3)	1.03509 (15)	0.70214 (10)	0.0150 (3)	
C1	0.4930 (4)	0.4251 (2)	0.62080 (14)	0.0255 (5)	
H1A	0.3570	0.4549	0.5897	0.038*	
H1B	0.4669	0.3374	0.6533	0.038*	
H1C	0.6414	0.4227	0.5771	0.038*	
C2	0.6840 (3)	0.48102 (18)	0.73647 (12)	0.0164 (4)	
C3	0.8446 (4)	0.37256 (18)	0.73332 (13)	0.0209 (4)	
H3	0.8404	0.3139	0.6916	0.025*	
C4	1.0113 (4)	0.34994 (19)	0.79132 (14)	0.0238 (4)	
H4	1.1196	0.2750	0.7897	0.029*	
C5	1.0204 (4)	0.4358 (2)	0.85130 (14)	0.0227 (4)	
H5	1.1355	0.4202	0.8905	0.027*	
C6	0.8611 (3)	0.54507 (19)	0.85434 (13)	0.0194 (4)	
H6	0.8684	0.6041	0.8956	0.023*	
C7	0.6917 (3)	0.56852 (17)	0.79755 (12)	0.0150 (4)	
C8	0.5446 (3)	0.78849 (18)	0.89132 (12)	0.0153 (4)	
C9	0.4431 (4)	0.73766 (19)	0.97760 (13)	0.0194 (4)	
H9	0.3236	0.6758	0.9856	0.023*	
C10	0.5126 (4)	0.7755 (2)	1.05209 (13)	0.0223 (4)	
H10	0.4427	0.7386	1.1104	0.027*	
C11	0.6845 (4)	0.8671 (2)	1.04128 (13)	0.0224 (4)	
H11	0.7347	0.8922	1.0920	0.027*	
C12	0.7825 (4)	0.92197 (19)	0.95603 (13)	0.0182 (4)	
H12	0.8974	0.9863	0.9485	0.022*	
C13	0.7135 (3)	0.88317 (17)	0.88174 (12)	0.0133 (4)	
C14	0.7201 (3)	0.99764 (17)	0.72442 (12)	0.0127 (3)	
H14	0.5569	0.9958	0.7172	0.015*	
C15	0.9046 (3)	1.05438 (17)	0.66405 (12)	0.0124 (3)	
C16	0.8998 (3)	1.12626 (17)	0.57456 (12)	0.0126 (4)	
C17	1.0842 (3)	1.20866 (18)	0.53447 (13)	0.0166 (4)	
H17	1.2131	1.2173	0.5651	0.020*	
C18	1.0791 (3)	1.27779 (19)	0.44998 (13)	0.0192 (4)	
H18	1.2047	1.3334	0.4233	0.023*	
C19	0.8925 (3)	1.26635 (19)	0.40436 (13)	0.0184 (4)	
H19	0.8903	1.3136	0.3465	0.022*	
C20	0.7089 (3)	1.18527 (18)	0.44379 (13)	0.0180 (4)	
H20	0.5802	1.1772	0.4129	0.022*	
C21	0.7127 (3)	1.11581 (18)	0.52828 (12)	0.0152 (4)	
H21	0.5861	1.0606	0.5547	0.018*	

### Atomic displacement parameters (Å^2^)

**Table:**

	*U*^11^	*U*^22^	*U*^33^	*U*^12^	*U*^13^	*U*^23^
Se	0.01551 (10)	0.01235 (10)	0.01832 (11)	−0.00120 (7)	−0.00576 (7)	−0.00192 (7)
O1	0.0237 (7)	0.0152 (7)	0.0177 (7)	−0.0024 (5)	−0.0049 (6)	−0.0032 (5)
N1	0.0117 (7)	0.0131 (7)	0.0145 (8)	−0.0021 (6)	−0.0051 (6)	−0.0002 (6)
N2	0.0108 (7)	0.0180 (8)	0.0185 (8)	−0.0012 (6)	−0.0056 (6)	−0.0015 (6)
N3	0.0125 (7)	0.0174 (8)	0.0155 (8)	−0.0012 (6)	−0.0040 (6)	−0.0011 (6)
C1	0.0338 (12)	0.0244 (11)	0.0210 (10)	−0.0045 (9)	−0.0057 (9)	−0.0090 (8)
C2	0.0193 (9)	0.0138 (9)	0.0147 (9)	−0.0045 (7)	0.0008 (8)	0.0010 (7)
C3	0.0252 (10)	0.0148 (9)	0.0200 (10)	−0.0012 (8)	0.0030 (8)	−0.0017 (8)
C4	0.0218 (10)	0.0165 (10)	0.0282 (11)	0.0032 (8)	0.0033 (9)	0.0028 (8)
C5	0.0182 (10)	0.0219 (10)	0.0265 (11)	0.0008 (8)	−0.0053 (9)	0.0021 (8)
C6	0.0182 (9)	0.0181 (9)	0.0218 (10)	−0.0020 (7)	−0.0031 (8)	−0.0024 (8)
C7	0.0148 (9)	0.0112 (8)	0.0168 (9)	−0.0014 (7)	0.0006 (7)	0.0014 (7)
C8	0.0142 (9)	0.0152 (9)	0.0168 (9)	0.0021 (7)	−0.0053 (7)	−0.0016 (7)
C9	0.0177 (9)	0.0185 (10)	0.0200 (10)	−0.0012 (7)	−0.0018 (8)	0.0020 (8)
C10	0.0228 (10)	0.0273 (11)	0.0139 (9)	0.0043 (8)	−0.0026 (8)	0.0031 (8)
C11	0.0243 (10)	0.0285 (11)	0.0154 (10)	0.0017 (8)	−0.0081 (8)	−0.0020 (8)
C12	0.0178 (9)	0.0205 (10)	0.0179 (10)	−0.0017 (7)	−0.0074 (8)	−0.0017 (8)
C13	0.0129 (8)	0.0135 (8)	0.0126 (9)	0.0018 (7)	−0.0029 (7)	0.0006 (7)
C14	0.0129 (8)	0.0135 (8)	0.0129 (9)	−0.0002 (7)	−0.0056 (7)	−0.0021 (7)
C15	0.0110 (8)	0.0123 (8)	0.0148 (9)	−0.0003 (6)	−0.0031 (7)	−0.0043 (7)
C16	0.0122 (8)	0.0121 (8)	0.0127 (9)	0.0015 (7)	−0.0007 (7)	−0.0022 (7)
C17	0.0139 (9)	0.0183 (9)	0.0183 (10)	−0.0031 (7)	−0.0036 (8)	−0.0022 (7)
C18	0.0178 (9)	0.0187 (9)	0.0191 (10)	−0.0047 (7)	0.0002 (8)	0.0018 (8)
C19	0.0199 (10)	0.0193 (9)	0.0136 (9)	0.0020 (8)	0.0000 (8)	0.0012 (7)
C20	0.0168 (9)	0.0208 (10)	0.0174 (10)	−0.0006 (8)	−0.0060 (8)	−0.0021 (8)
C21	0.0134 (9)	0.0160 (9)	0.0163 (9)	−0.0032 (7)	−0.0023 (7)	−0.0018 (7)

### Geometric parameters (Å, º)

**Table:**

Se—C8	1.9202 (19)	C8—C13	1.400 (2)
Se—C7	1.9224 (19)	C9—C10	1.388 (3)
O1—C2	1.368 (2)	C9—H9	0.9500
O1—C1	1.434 (2)	C10—C11	1.387 (3)
N1—C14	1.351 (2)	C10—H10	0.9500
N1—N2	1.365 (2)	C11—C12	1.387 (3)
N1—C13	1.433 (2)	C11—H11	0.9500
N2—N3	1.313 (2)	C12—C13	1.388 (3)
N3—C15	1.369 (2)	C12—H12	0.9500
C1—H1A	0.9800	C14—C15	1.378 (2)
C1—H1B	0.9800	C14—H14	0.9500
C1—H1C	0.9800	C15—C16	1.467 (2)
C2—C3	1.388 (3)	C16—C21	1.392 (3)
C2—C7	1.404 (3)	C16—C17	1.402 (2)
C3—C4	1.389 (3)	C17—C18	1.390 (3)
C3—H3	0.9500	C17—H17	0.9500
C4—C5	1.380 (3)	C18—C19	1.385 (3)
C4—H4	0.9500	C18—H18	0.9500
C5—C6	1.390 (3)	C19—C20	1.388 (3)
C5—H5	0.9500	C19—H19	0.9500
C6—C7	1.388 (3)	C20—C21	1.390 (3)
C6—H6	0.9500	C20—H20	0.9500
C8—C9	1.393 (3)	C21—H21	0.9500
			
C8—Se—C7	96.32 (8)	C11—C10—C9	119.82 (18)
C2—O1—C1	117.14 (16)	C11—C10—H10	120.1
C14—N1—N2	110.78 (14)	C9—C10—H10	120.1
C14—N1—C13	130.06 (15)	C12—C11—C10	119.69 (19)
N2—N1—C13	118.87 (15)	C12—C11—H11	120.2
N3—N2—N1	106.67 (14)	C10—C11—H11	120.2
N2—N3—C15	109.61 (14)	C11—C12—C13	120.24 (18)
O1—C1—H1A	109.5	C11—C12—H12	119.9
O1—C1—H1B	109.5	C13—C12—H12	119.9
H1A—C1—H1B	109.5	C12—C13—C8	120.86 (16)
O1—C1—H1C	109.5	C12—C13—N1	116.82 (16)
H1A—C1—H1C	109.5	C8—C13—N1	122.32 (16)
H1B—C1—H1C	109.5	N1—C14—C15	104.97 (16)
O1—C2—C3	125.05 (17)	N1—C14—H14	127.5
O1—C2—C7	114.90 (17)	C15—C14—H14	127.5
C3—C2—C7	120.05 (19)	N3—C15—C14	107.96 (16)
C2—C3—C4	119.93 (19)	N3—C15—C16	122.74 (15)
C2—C3—H3	120.0	C14—C15—C16	129.29 (17)
C4—C3—H3	120.0	C21—C16—C17	118.71 (17)
C5—C4—C3	120.32 (19)	C21—C16—C15	121.23 (16)
C5—C4—H4	119.8	C17—C16—C15	120.06 (17)
C3—C4—H4	119.8	C18—C17—C16	120.19 (18)
C4—C5—C6	120.0 (2)	C18—C17—H17	119.9
C4—C5—H5	120.0	C16—C17—H17	119.9
C6—C5—H5	120.0	C19—C18—C17	120.64 (17)
C7—C6—C5	120.45 (19)	C19—C18—H18	119.7
C7—C6—H6	119.8	C17—C18—H18	119.7
C5—C6—H6	119.8	C18—C19—C20	119.46 (18)
C6—C7—C2	119.22 (18)	C18—C19—H19	120.3
C6—C7—Se	125.27 (14)	C20—C19—H19	120.3
C2—C7—Se	115.50 (15)	C19—C20—C21	120.26 (18)
C9—C8—C13	117.89 (17)	C19—C20—H20	119.9
C9—C8—Se	117.98 (14)	C21—C20—H20	119.9
C13—C8—Se	123.97 (14)	C20—C21—C16	120.73 (17)
C10—C9—C8	121.44 (18)	C20—C21—H21	119.6
C10—C9—H9	119.3	C16—C21—H21	119.6
C8—C9—H9	119.3		
			
C14—N1—N2—N3	−0.75 (19)	C9—C8—C13—N1	−177.53 (17)
C13—N1—N2—N3	−175.16 (14)	Se—C8—C13—N1	7.1 (3)
N1—N2—N3—C15	0.31 (19)	C14—N1—C13—C12	−133.19 (19)
C1—O1—C2—C3	2.6 (3)	N2—N1—C13—C12	40.0 (2)
C1—O1—C2—C7	−178.26 (16)	C14—N1—C13—C8	46.3 (3)
O1—C2—C3—C4	179.81 (17)	N2—N1—C13—C8	−140.54 (18)
C7—C2—C3—C4	0.7 (3)	N2—N1—C14—C15	0.86 (19)
C2—C3—C4—C5	−0.9 (3)	C13—N1—C14—C15	174.46 (17)
C3—C4—C5—C6	0.4 (3)	N2—N3—C15—C14	0.2 (2)
C4—C5—C6—C7	0.2 (3)	N2—N3—C15—C16	179.29 (16)
C5—C6—C7—C2	−0.5 (3)	N1—C14—C15—N3	−0.65 (19)
C5—C6—C7—Se	−179.52 (14)	N1—C14—C15—C16	−179.64 (17)
O1—C2—C7—C6	−179.22 (16)	N3—C15—C16—C21	162.95 (17)
C3—C2—C7—C6	0.0 (3)	C14—C15—C16—C21	−18.2 (3)
O1—C2—C7—Se	−0.1 (2)	N3—C15—C16—C17	−17.9 (3)
C3—C2—C7—Se	179.15 (13)	C14—C15—C16—C17	161.00 (18)
C13—C8—C9—C10	−2.4 (3)	C21—C16—C17—C18	−0.2 (3)
Se—C8—C9—C10	173.23 (15)	C15—C16—C17—C18	−179.43 (17)
C8—C9—C10—C11	0.9 (3)	C16—C17—C18—C19	0.0 (3)
C9—C10—C11—C12	1.1 (3)	C17—C18—C19—C20	0.2 (3)
C10—C11—C12—C13	−1.5 (3)	C18—C19—C20—C21	−0.2 (3)
C11—C12—C13—C8	0.0 (3)	C19—C20—C21—C16	0.0 (3)
C11—C12—C13—N1	179.48 (17)	C17—C16—C21—C20	0.2 (3)
C9—C8—C13—C12	1.9 (3)	C15—C16—C21—C20	179.44 (17)
Se—C8—C13—C12	−173.39 (15)		

### Hydrogen-bond geometry (Å, º)

Cg1 is the centroid of the C1–C6 ring.

**Table:**

*D*—H···*A*	*D*—H	H···*A*	*D*···*A*	*D*—H···*A*
C18—H18···O1^i^	0.95	2.54	3.472 (2)	165
C14—H14···N3^ii^	0.95	2.58	3.520 (2)	170
C10—H10···*Cg*1^iii^	0.95	2.82	3.630 (2)	144

Symmetry codes: (i) −*x*+2, −*y*+2, −*z*+1; (ii) *x*−1, *y*, *z*; (iii) −*x*+1, −*y*+1, −*z*+2.
