# Supplementary figures and images for: Crystal structure of 1-{2-[(2-meth­oxy­phen­yl)selan­yl]phen­yl}-4-phenyl-1H-1,2,3-triazole
Source: Acta Crystallogr E Crystallogr Commun. 2015 Feb 25;71(Pt 3):o202–3. doi: 10.1107/S2056989015003230 (PMC4350762; doi:10.1107/S2056989015003230)

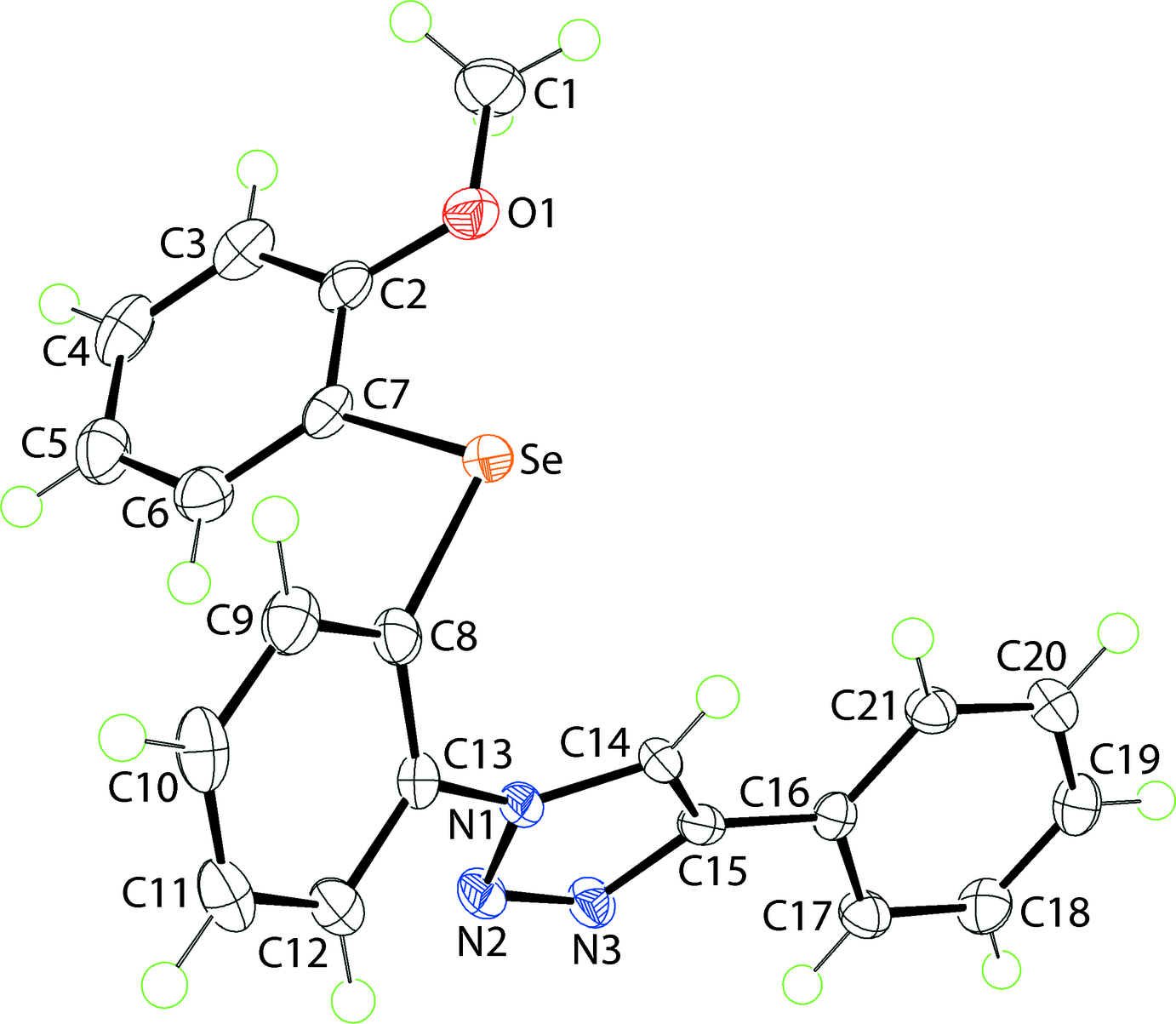

Supplement: Supplementary file 4 [file e-71-0o202-fig1.tif]

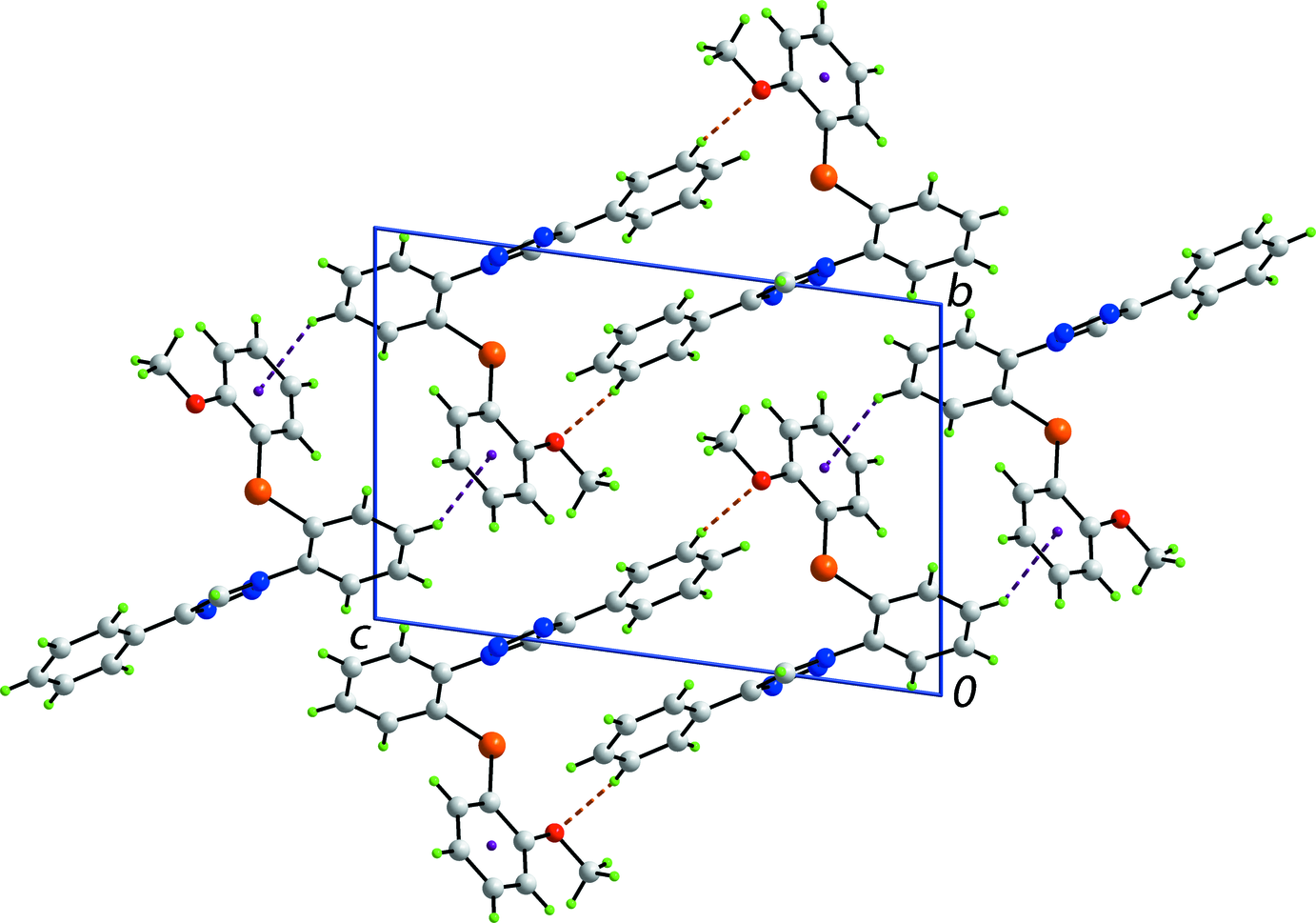

Supplement: Supplementary file 5 [file e-71-0o202-fig2.tif]
